# Multidomain Social Determinants of Depressive Symptoms for the Elderly with Chronic Diseases: Evidence from the China Health and Retirement Longitudinal Study

**DOI:** 10.3390/healthcare9121765

**Published:** 2021-12-20

**Authors:** Tao Zhang, Xiaohe Wang, Yongjian Xu

**Affiliations:** 1 Department of Health Policy and Management, School of Public Health, Hangzhou Normal University, Hangzhou 311121, China; 20210085@hznu.edu.cn (T.Z.); 20021200@hznu.edu.cn (X.W.); 2 School of Public Policy and Administration, Xi’an Jiaotong University, Xi’an 710048, China

**Keywords:** depression, elderly, chronic diseases, social determinants, China

## Abstract

Elderly individuals with chronic diseases (CDs) have a much higher risk of mental disorders, especially depression. This study aimed to identify the multidomain social determinants of occurrence and degree of depressive symptoms for the Chinese elderly with CDs. Data of 3438 elderly individuals (aged over 60 years) with CDs were drawn from the fourth wave of the China Health and Retirement Longitudinal Study implemented in 2018. Logistic regression was used to describe associations with the occurrence of depressive symptoms within and across multidomain social determinants (demographic, economic, neighborhood, environmental, and social and cultural). The Shapley value decomposition method was used to measure the relative importance of variables of the five domains. A quantile regression model was used to test how the effects of social factors vary across different points of depression score distributions. Approximately 40.1% of Chinese elderly individuals with CDs reported depressive symptoms. Respondents who were female, had a low income, experienced a disability, lived in rural areas, and were not engaged in work had a higher probability of suffering from depressive symptoms. Conversely, increased age, being covered by social security and being well-educated had a protective effect. Data also showed that the effects of these associated factors varied across different points of depression score distributions. The fact that socially disadvantaged people are more vulnerable to severe depressive symptoms implies that psychological health services and intervention strategies should target this population.

## 1. Introduction

With the growing number of individuals reaching old age, the population is rapidly aging worldwide. China is no exception. The percentage of people aged 60 years and over in China jumped from 10.5% in 2000 to 18.7% in 2020, and is predicted to reach 34.5% by 2050 [1,2]. The aging of the population has brought enormous challenges to the Chinese healthcare system. In the first place, chronic diseases (CDs) are a problem that cannot be ignored due to the fact that more than 70% of the elderly are diagnosed with at least one CD [3]. CDs have been considered as the dominant cause of premature death of the elderly.

In recent years, the Chinese government has launched a series of policies to improve the physiological function of elderly patients with CDs. For example, the elderly with CDs are provided health check-ups twice a year free of charge [4]. However, the Chinese government has paid less attention to the mental health of these individuals. Previous studies have documented that the elderly with CDs have a much higher risk of mental disorders than the general population, especially depression [5,6]. In China, approximately 42% of the elderly suffering from CDs were diagnosed with depression, which is much higher than that of the general population aged over 60 years (17%) [7,8]. Moreover, existing evidence also found that several CDs (e.g., hypertension, diabetes, and strokes) were strongly associated with depression [5,6,9]. Hence, practitioners and policy makers should make effective interventions for these elderly people with CDs.

Depression, one of the most common problems of old age in the psychological context, can impair the health status, create a risk of reduced quality of life for patients and their family members, and even lead to suicide [10]. According to the World Health Organization (WHO), depression is supposed to be one of the major determinants causing premature deaths and disability in the world, and it ranks second after ischemic heart disease in terms of disease burden after decades [11,12]. Therefore, understanding the possible risk factors associated with depression amongst the elderly people and implementing targeted interventions, particularly in China with accelerated aging and high incidence of CDs, are greatly important.

Although depression is influenced by numerous interwoven biological and social mechanisms that exist in various domains [13], social determinants including marital status, education, and income have consistently been identified as important factors associated with the occurrence of depression in contemporary available literatures [14,15]. For example, age was found to be associated with depression, and interestingly, this association is inverse or U-shaped in several studies [16,17]. A higher socioeconomic status is often associated with a lower likelihood of depression, especially for people with a high educational level and income [18,19]. Additionally, some other research focused on personal lifestyle and healthy behavior. For instance, Zhou et al. reported that sleep disorders and limited physical activity were important risk factors for the occurrence of depression amongst patients with chronic respiratory diseases [6]. In addition, alcohol drinking, smoking, and BMI were found to be associated with depression symptoms in published literature [15].

Multidomain social determinants of depression have been extensively explored in previous studies, but there are still some gaps. Firstly, most studies mainly analyzed factors of depression from a single aspect, such as economic status and social capital [20,21]. Consequently, knowledge about a comprehensive set of social determinants on depression amongst the elderly in China, especially those with CDs, is scarce. Secondly, evidence from national investigations is lacking because previous studies adopted data from a single province of mainland China [22,23]. Thirdly, whether social determinants have different effects at different levels of depression remains unknown because most studies paid more attention to factors influencing the occurrence of depression, rather than the degree of depression.

To fill these gaps, this study aimed to examine multidomain social determinants for depressive symptoms amongst the elderly with CDs using national data in China. Furthermore, we explored the differentiated (relative) importance of these factors depending on the level of depression score distributions.

### Conceptual Framework—Multidomain Social Determinants of Mental Disorders

**Figure 1:**
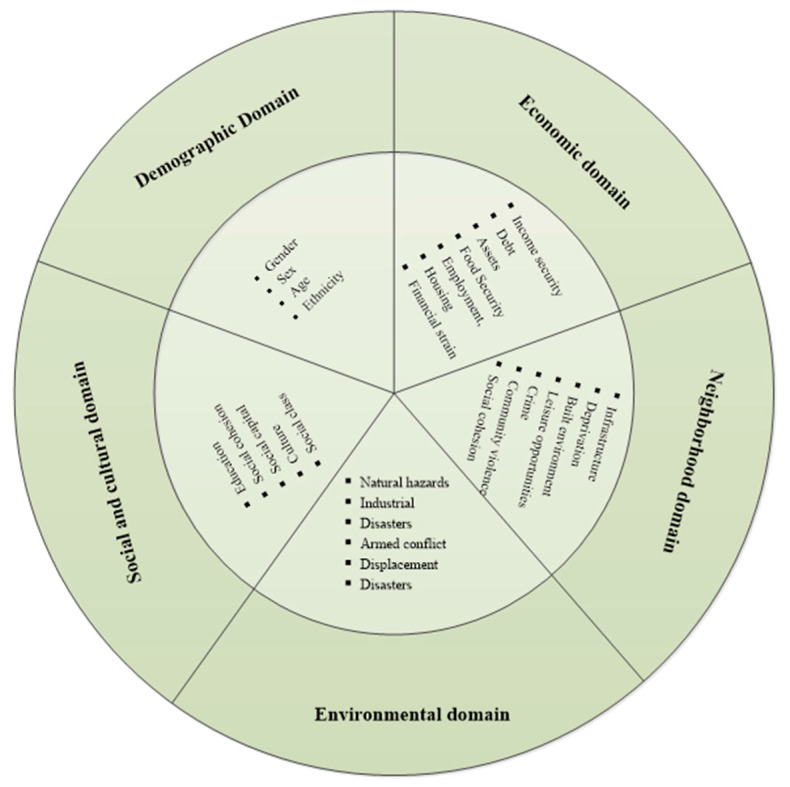
Conceptual framework for multiple social determinants domains of mental disorders.

Our study adopted the conceptual framework developed by Lund et al. as a conceptual tool for understanding the potential factors associated with depression amongst the elderly with CDs [13]. This conceptual framework identified the key domains of the social determinants of mental disorders on the basis of previous conceptual work and the WHO Commission on Social Determinants of Health frameworks, namely, demographic, economic, neighborhood, environmental events, and social and cultural domains. The framework integrated multidimensional social determinants and their interaction so as to provide a comprehensive explanation on mental disorders (Figure 1).

In the demographic domain, the specific demographic characteristics (such as gender, age, ethnicity, and life expectancy) of populations convey risk for, or protection from, mental illness [24]. The economic domain contains factors related to the production, consumption, and transfer of wealth that convey risk for, or protection from, mental illness, such as income, debt, assets, food security, employment status, housing, income inequality, macroeconomic trends, and subjective financial strain. The neighborhood domain constitutes constellations of environmental risk factors, such as safety, deprivation at the neighborhood level, access to recreational facilities, and availability of services. The effects of the neighborhood risk factors still remain even after controlling for individual socioeconomic deprivation and other exposures, whereas these factors frequently interact with coexisting individual factors [25]. Environmental events are defined as serious disruptions of the functioning of a community that exceed its ability to cope by use of its own resources and convey risk for mental illness [13]. The risk factors for mental disorders in the context of environmental events can be summarized on the basis of previous studies, such as a higher level of exposure to the event, previous disability experience, low socioeconomic status, insecurity, and violence [26,27,28]. In terms of the social and cultural domain, it encompasses education, family and peer relationships, social capital, social networks, culture, and group membership. These factors affect individual mental health via the pathway potentially acting on cognitive reserve, self-efficacy, social skills, social capital, social support, and trust [29,30,31].

## 2. Materials and Methods

### 2.1. Sample

This study analyzed the data from the fourth wave of the China Health and Retirement Longitudinal Study (CHARLS). CHARLS is a biennial survey conducted by the China Center for Economic Research at Peking University with the aim of collecting a high-quality, nationally representative sample of Chinese residents aged 45 years and older. A multistage sampling strategy was adopted in CHARLS. At the first stage, all county-level units were sorted (stratified) by region, within region by urban district or rural county, and by GDP per capita (Tibet was the only province not included). A region was a categorical variable based on the province area. After sorting (stratification), 150 counties or urban districts were chosen with probability proportional to the population size. For each county-level unit, three villages and urban neighborhoods were randomly chosen with probability proportional to the population [32].

The data of CHARLS include information on basic demographics, family, health status, healthcare and health insurance, employment, and household economy of the respondents and their living spouses. In the fourth wave implemented in 2018, the survey sample consisted of 19,816 individuals over 45 years old and their spouses from 12,400 households in 450 village-level units and 150 county-level units [33]. More details about the sampling method and the questionnaire can be found elsewhere [32]. The CHARLS data can be accessed through its official website (http://charls.pku.edu.cn/pages/data/2018-charls-wave4/zh-cn.html) (accessed on 28 April 2019).

**Figure 2:**
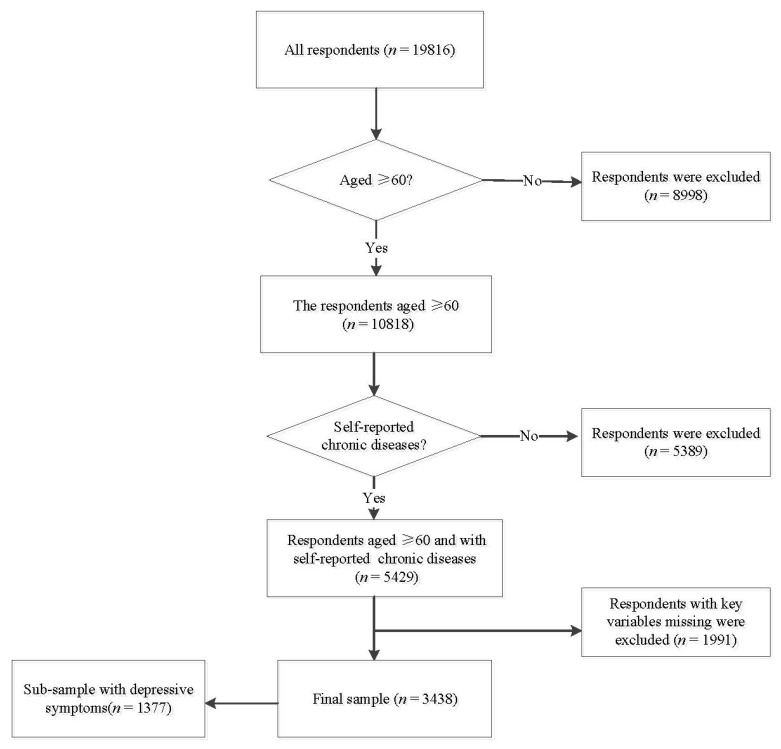
The flow diagram for sample screening.

In this study, a total of 5429 respondents aged over 60 years old and with self-reported CDs were included. After excluding respondents with missing key variables, 3438 were left for data analysis. Additionally, a subsample of 1377 respondents with depression were selected from a final sample to analyze the associations of multidomain social determinants with degrees of depression. More details can be found in Figure 2.

### 2.2. Outcome Variables

Depressive symptoms were identified using the 10-item Center for Epidemiologic Studies Depression Scale (CESD-10), which has high validity and reliability in the Chinese population [1,34]. Participants were asked about the number of days they experienced every item in the past week. Respondents reported the frequency of occurrence of eight negative effect items on a four-point scale: 0 (rarely or none of the time; less than 1 day), 1 (some of the time; 1–2 days), 2 (much or a moderate amount of the time; 3–4 days), or 3 (most or all the time; 5–7 days). For the two positively worded items, the answers were reverse-coded. The sum of the CESD-10 scores ranged from 0 to 30, and a score of 10 was used as the cut-off point to identify those people who suffered from depressive symptoms or not [35].

In this study, scores over 10 and less than 10 were coded as 1 and 0, respectively, and as the first outcome variable. Additionally, we selected a score ≥10 as the second outcome variable to explore impacts of multiple variables on depressive symptoms at different levels.

### 2.3. Independent Variables

On the basis of data availability and the conceptual framework developed by Lund et al. [13], a total of 12 social-related variables were selected from CHARLS followed by prior empirical investigations [36,37,38]. The factors in this study were divided into five domains: demographic, economic, neighborhood, environmental event, and social and cultural. Domain definitions from the Lund study and variable construction are provided in Supplemental Table S1.

The demographic domain included gender, age, and marriage status. The economic domain included annual income and working status. The neighborhood domain included residence and access to physical examination. The environmental domain included working age and disability experience. Finally, the social and cultural domain included education level, living family house, and amount of social security.

### 2.4. Data Analysis

This study adopted a two-step analysis strategy. Firstly, a logistic regression model was used to determine factors associated with the occurrence of depressive symptoms. Then, the Shapley value decomposition was adopted to identify the contributions of variables of the five domains to the outcome variable. Secondly, we established quantile regression models to further explore the change in the impact of multidomain social determinants on varying depression scores. In order to address the potential bias resulting from excluding samples with missing data, a bootstrapping strategy was adopted for building the regression model.

#### 2.4.1. Stage One: Logistic Regression Model and Shapley Value Decomposition

A logistic regression model was used to determine the multivariable associations of depressive symptoms with variables of the five domains. Additionally, the standardized coefficient size in a regression model is frequently used to identify which main predictors are relatively more important for the occurrence of depression. However, health research often involves inherently imprecise measures of complex concepts, which result in bias using coefficients to determine the relative importance of independent variables. In accordance with previous studies [39,40], we adopted the Shapley value regression to identify the relative importance of variables of the five domains.

The Shapley values measure the extent to which an outcome varies when an independent variable is added to different, pre-existing sets of variables. The change in the marginal effect to R^2^ after adding a predictor represents the proportion of contribution influenced by that predictor [41]. The Shapley value of a single attribute $x_{j}$ with a simplified notation can be given by:

$$SV_{j}=\sum_{k}^{}\sum_{k}^{}\gamma_{k}\left[v(M_{i|j})-v(M_{i|j(-j)})\right]$$

where $SV_{j}$ is the Shapely value for predictor *j*, $v\left(M_{i|j}\right)$ is the R^2^ of a model containing predictor *j*, $v\left(M_{i|j\left(-j\right)}\right)$ is the R^2^ of the same model i without *j*, and $\gamma_{k}$ is a weight based on the total number of predictors in the model.

#### 2.4.2. Stage Two: Quantile Regression Models

Quantile regression models were applied to analyze the extent to which the impact of social factors vary depending on the depression score distribution using data of respondents with depressive symptoms (CESD-10 scores ≥10). Unlike the ordinary least square describing the relationship between dependent and independent variables using the conditional mean alone, this approach allows the estimation of an ensemble of models for conditional quantile functions, which is useful for analyzing the effects of predictors at different levels of the outcome distribution [42].

The specific quantile of the outcome distribution, conditioned to the values of the predictor variables is given by:

$$Q_{yi}\left[\tau |x_{1i},x_{2i},\ldots ,x_{ki}\right]=\beta_{0}\left(\tau \right)+\beta_{1}\left(\tau \right)x_{1i}+\beta_{2}\left(\tau \right)x_{2i}+\cdots +\beta_{k}\left(\tau \right)x_{ki}$$

where $Q_{yi}\left(\tau |\right)$ denotes the *τ*-th quantile of the conditional distribution of $y_{i}$, the outcome variable, with 0 < *τ* < 1. Therefore, the regression parameter $\beta_{k}\left(\tau \right)$ denotes how the specified quantile changes with one-unit change in $x_{k}$. The parameter vector $\beta_{k}\left(\tau \right)$ is estimated to solve a minimum problem; it can be formulated as a linear programming problem and calculated efficiently [39,43].

In this study, we selected the 10th, 50th, and 90th conditional quantiles to analyze the impacts of independent variables on mild, moderate, and severe depression, respectively. The variance–covariance matrix was estimated by bootstrap with 400 replications in the process of performing simultaneous quantile regressions. All analyses were performed on STATA14.0.

## 3. Results

### 3.1. Characteristics of Respondents

Table 1 shows the characteristics of respondents. Of the 3438 old adults, 40.1% respondents reported that they had depressive symptoms, and the average CESD-10 score for respondents who suffered from depressive symptoms was 16.1. In demographic characteristics, males and females accounted for 56.2% and 43.8%, respectively. The average age of the respondents was 68.6 years. Most respondents (70.8%) were married. In the economic domain, a small part of the elderly (8.1%) earned more than 20,000 CNY a year, and 88.3% respondents were not working at present. In terms of the neighborhood domain, 81.5% respondents lived in rural areas, and approximately one third of the elderly underwent a physical examination in the past two years. The vast majority (97.6%) lived in the family house, and 43.8% respondents reported that they suffered disabilities. In terms of educational level, nearly half of the respondents (48.7%) graduated from primary school.

### 3.2. Multidomain Social Determinants of Depressive Symptoms: Results from Logistic Regression and Shapley Value Regression

Table 2 reports the association between multidomain social determinants and the occurrence of depressive symptoms amongst the elderly with CDs and provides the contribution percentage of variables of the five domains to depressive symptoms on the basis of the Shapley value regression.

In the demographic domain, female respondents (*β* = 0.606, 95%CI: 0.436 to 0.777) and those with another marriage status (*β* = 0.221, 95%CI: 0.034 to 0.408) were more likely to report depressive symptoms. An increased age (*β* = −0.027, 95%CI: −0.041 to −0.013) appeared to be associated with lower CES-D10 scores. The annual income (*β* = 0.437, 95%CI: 0.023 to 0.897) in the economic domain was found to be negatively associated with depressive symptoms. Those who were not engaged in work (*β* = 0.249, 95%CI: 0.004 to 0.495) had an increased risk of depression. A residence located in rural areas (*β* = 0.235, 95%CI: 0.011 to 0.460) was associated with an increased risk of depressive symptoms. Elderly people experiencing disabilities were more likely to become depressed (*β* = 0.457, 95%CI: 0.303 to 0.612). In the social and cultural domain, respondents with a higher educational level (*β* = −0.417, 95%CI: −0.738 to −0.204) were unlikely to report a depressive symptom. Additionally, elderly covered by social security (*β* = −0.181, 95%CI: −0.343 to −0.019) had reduced risk of depressive symptoms.

The results of the Shapley value regression analysis reveal that the variables related to sociodemographic, social and cultural, economic, neighborhood, and environmental factors contributed 33.5%, 28.9%, 26.5%, 10.8%, and 1.1% to the total variance, respectively.

### 3.3. Association of Social Determinants on Varying Degrees of Depressive Symptoms: Results from Quantile Regressions

The quantile regression results using data from respondents with depressive symptoms (CESD−10 scores ≥ 10) are summarized in Table 3. The coefficients of variables varied across the three quantiles (10th, 50th, and 90th), indicating that the association of multi-domain social determinants with depressive symptoms depends on its distribution level.

Income, working status, rural/urban residence, working age, living with family members, and amount of social security were not associated with depressive symptoms in any of the quantiles of the elderly with depression. In the lowest quantile (10th), only the female gender was associated with depressive symptoms (*β* = 0.489, 95%CI: 0.010 to 0.967), but the number of factors increased with higher depression scores. At the 50th quantile, depressive symptoms were most strongly associated with being unmarried (*β* = 0.971, 95%CI: 0.030 to 1.913), followed by the female gender (*β* = 0.604, 95%CI: 0.143 to 1.865) and, lastly, having a disability (*β* = 0.100, 95%CI: 0.093 to 1.806). For those in the highest quantile of depressive symptoms, in addition to being unmarried (*β* = 0.651, 95%CI: 0.881 to 3.222) and having a disability (*β* = 0.259, 95%CI: 0.008 to 2.511), a younger age and low education were significantly associated with depressive symptoms in the elderly with CDs.

## 4. Discussion

This study sheds some lights on the multidomain social determinants of depressive symptoms amongst the elderly with CDs using data from a nationwide survey in China. Overall, depressive symptoms were associated with demographic (gender, age, and marriage status), economic (annual income and working status), neighborhood (residence), environmental (disability experience), and social and cultural (education and social security) determinants in the fully adjusted model.

The results show that the prevalence rates for depressive symptoms of elderly patients with CDs reached 40.1%, which is higher than 32.5% amongst the general elderly population [1]. Two reasons can explain such a high rate of occurrence. On the one hand, physiological pain caused by CDs can result in stress and anxiety to a great extent. Compared with elderly people without CDs, a higher risk of depressive symptoms in elderly people with CDs may also be related to the high economic burden of disease demonstrated by previous study [44]. On the other hand, preventive measures aimed at alleviating depressive disorders were seldom conducted in China, despite the fact that many health promotions projects, such as free physical check-ups for elderly people, have been implemented in recent years [4]. Therefore, depressive symptoms are predicted to be a serious public health issue in China, especially for the accelerating aging population.

Consistent with previous studies [36,45], our findings suggest that women suffered from a higher risk of depression. Furthermore, quantile regressions indicated that women experienced a higher degree of depressive symptoms compared with men. This result is attributed to the fact that females in traditional Chinese society take on more responsibility in looking after the family and are in a socially disadvantaged position [46]. Projects aiming to improve women’s mental health need to be implemented in the near future. Surprisingly, we observed that age was a protective factor for depressive symptoms amongst the elderly, and this protective effect was stronger amongst patients with severe depression, which is inconsistent with a previous study [1]. Our study objects, unlike other studies, focused on elderly people aged over 60 with CDs. Elderly people can usually receive more respect and support from the family and society in traditional Chinese culture. Moreover, these elderly people are generally retired and have less pressure in their work and lives compared with their younger counterparts. In addition to the above demographic factors, the marriage status was also demonstrated to be significantly associated with depression. Elderly people who are married and live with their spouse can receive help materially and spiritually, thus further reducing financial hardship and loneliness. By contrast, CD patients who are widowed or separated are unable to receive long-term care from their spouse, which causes a low quality of life, poor physical and mental health, and reduced social interaction and life satisfaction [47,48].

Education has often been linked with depressive symptoms with advanced education being associated with a low prevalence of depressive symptoms [18,46]. The findings of the present study are not an exception. Conversely, we also observed that the protective effect of being well-educated appears to be stronger for people with severe depression. On the one hand, the level of education can be viewed in the context of an individual’s capacity to communicate and receive health information [49]. A higher education is believed to affect the brain, creating cognitive reserve and resilience against mental disorders. Hence, CD patients with a high education are more informed about their conditions and self-health management, thereby reducing the risk of suffering from severe depression. On the other hand, education may reduce depressive symptoms through a potential mechanism of increasing social capital and social support [50]. Being less educated has been found to be linked to reduced social cohesion, poor social capital, and chronic stress, increasing the risk of common mental disorders [29,31].

Similar to another study [1], people without work had a higher possibility to report depressive symptoms, and these people were more likely to suffer from severe depression. Generally, unemployment means losing stable income due to the lack of universal coverage of social security in China. Decreased income further limits personal consumption of healthcare services and relaxing entertainment. Importantly, elderly adults who are not working may also have fewer social contacts and also reduced opportunities to widen their social network and relationships [39]. Previous studies have shown that more intimate social engagement encourages much greater neurostimulation, and neurotransmitter stimulation is well known to improve overall mood [51,52]. Thus, being employed may decrease depressive symptoms via the pathways of neurostimulation.

Elderly adults covered by social security were found to have less risk of depression. The main reason for this result can be attributed to personal socioeconomic status. Elderly people with pension insurance have a better material security in their lives in China. Moreover, the finding of the negative association of depressive symptoms with income also reflects a protective effect of socioeconomic status. That is to say, the advantage in economy can prevent the occurrence of depressive symptoms in the elderly with CDs. Similarly, the great disparity between rural and urban areas in economic development and access to healthcare services in China obviously explained why the elderly living in rural areas are more likely to suffer from depressive symptoms [53,54].

Experiencing disability in the environmental domain made a relatively large contribution to the variance of depressive symptoms on the basis of the results of the Shapley value regression, implying that this variable is closely associated with the occurrence of depression. Indeed, it is not difficult to understand that physical illnesses have a negative impact on mental health due to the fact that these patients are limited in performing activities of daily living and routine social activities [55]. Additionally, the effect of this risk factor appears to become stronger with a high degree of depression. Accordingly, intervention policies related to mental health should give priority to elderly people with impaired physiological functions.

Some limitations in this study should be acknowledged. Firstly, causal conclusions cannot be reached due to the fact that the data we used were collected from a cross-sectional survey. Secondly, even though we selected as many variables as possible, the independent variables that we selected were limited to reflect comprehensive, multidomain social determinants of depressive symptoms. Thirdly, recall bias might exist in our study because the data employed in our study were self-reported. Also, potential bias caused by the selection of sample needs to be considered.

Given these limitations, we have extended the existing research by focusing on the elderly population with CDs using nationally representative sample. Moreover, unlike other studies, this analysis provided more detailed information about the association of social related factors with depressive symptoms across different distribution levels.

## 5. Conclusions

The present study revealed a high prevalence of depressive symptoms amongst the elderly with CDs in China. Overall, the depressive symptoms are more frequent in socially disadvantaged populations, and social/family support and economic factors are great for reducing the degree of depressive symptoms in this population. However, the effects of these factors vary depending on the level of depressive symptoms distributions. How psychological health services and targeted policy interventions can be delivered effectively to elderly patients with CDs in China need to be considered. For example, cooperation should be encouraged between general practitioners and psychiatrists to strengthen the health management for the elderly with CDs; the coverage and welfare of social security should be expanded to reduce financial hardship of low-income groups; regular mental health education should be organized; and psychological counselling service should be provided free of charge for elderly people.

## Figures and Tables

**Table 1:** Characteristics of respondents.

Domain	Variables	*n*/Mean	%/S.D.
Demographic	Gender		
	Male	1932	56.2
	Female	1506	43.8
	Age (year)	68.6	6.4
	Marriage status		
	Married	2435	70.8
	Others	1003	29.1
Economic	Annual income (CNY)		
	<20,000	3159	91.9
	≥20,000	279	8.1
	Working status		
	Working	403	11.7
	Not working	3035	88.3
Neighborhood	Residence		
	Urban	637	18.5
	Rural	2801	81.5
	Access to physical examination		
	Yes	1179	34.3
	No	2259	65.7
Environmental events	Working age (year)		
	<18	3159	91.9
	≥18	279	8.1
	Disability experience		
	No	1932	56.2
	Yes	1506	43.8
Social and cultural	Education		
	Illiteracy	961	28.0
	Primary school	1675	48.7
	Middle school or above	802	23.3
	Living in family house		
	Yes	3354	97.6
	No	84	2.4
	Amount of social security		
	No	1141	33.2
	One	2087	60.7
	Two or above	210	6.1
Outcome 1	Occurrence of depressive symptoms		
	No	2061	59.9
	Yes	1377	40.1
Outcome 2	Degree of depressive symptoms	16.1	4.9

**Table 2:** Results from logistic regression and Shapley value regression (*n* = 3438).

Domain	Variables	*β*	*p*	95%CI	Shapley Value	Contribution to R^2^ (%)
Demographic	Gender (ref. = male)				0.0224	33.52
	Female	0.606	<0.001	(0.436, 0.777)		
	Age	−0.027	<0.001	(−0.041, −0.013)		
	Marriage status (ref. = married)					
	Others	0.221	0.020	(0.034, 0.408)		
Economic	Annual income (ref. = ≥20,000)				0.0178	26.53
	<20,000	0.437	0.041	(0.023, 0.897)		
	Working status (ref. = working)					
	Not working	0.249	0.046	(0.004, 0.495)		
Neighborhood	Residence (ref. = urban)				0.0067	10.81
	Rural	0.235	0.040	(0.011, 0.460)		
	Access to physical examination (ref. = yes)					
	No	0.014	0.954	(−0.472, 0.501)		
Environmental event	Working age (ref. = ≥18)				0.0007	1.06
	<18	0.079	0.381	(−0.098, 0.256)		
	Disability experience (ref. = no)					
	Yes	0.457	<0.001	(0.303, 0.612)		
Social and cultural	Education (ref. = illiteracy)				0.0193	28.81
	Primary school	−0.054	0.609	(−0.264, 0.155)		
	Middle school or above	−0.471	0.001	(−0.738, −0.204)		
	Living in family house (ref. = yes)					
	No	0.225	0.512	(−0.448, 0.899)		
	Amount of social security (ref. = none)					
	One	−0.181	0.029	(−0.343, −0.019)		
	Two or above	0.037	0.833	(−0.311, 0.386)		
χ^2^		507.271	<0.001			
R^2^		0.281				

**Table 3:** Quantile regression results on the 10th, 50th, and 90th quantiles of depressive symptoms (*n* = 1377).

Variables	Q10		Q50		Q90	
	*β*	95%CI	*β*	95%CI	*β*	95%CI
Gender (ref. = male)						
Female	0.489 *	(0.010, 0.967)	0.604 *	(0.143, 1.865)	−0.144	(−1.508, 1.220)
Age	0.005	(−0.025, 0.036)	−0.018	(−0.088, 0.501)	−0.092 *	(−0.177, −0.007)
Marriage status (ref. = married)						
Others	0.087	(−0.335, 0.510)	0.971 *	(0.030, 1.913)	0.651 *	(0.081, 3.222)
Annual income (ref. = ≥20,000)						
<20,000	0.203	(−0.278, 0.684)	−0.561	(−1.467, 0.346)	0.201	(−1.290, 1.691)
Working status (ref. = working)						
Not working	−0.280	(−0.744, 0.183)	−0.876	(−2.226, 0.473)	−0.977	(−3.151, 1.197)
Residence (ref. = urban)						
Rural	−0.159	(−0.874, 0.556)	−0.077	(−1.156, 1.002)	0.589	(−0.825, 2.004)
Access to physical examination (ref. = yes)						
No	−0.296	(−1.657, 1.063)	−0.366	(−3.016, 2.282)	−0.308	(−6.711, 2.093)
Working age (ref. = ≥18)						
<18	0.34	(−0.848, 1.529)	0.021	(−5.074, 5.116)	−0.488	(−6.242, 1.265)
Disability experience (ref. = no)						
Yes	0.258	(−0.115, 0.632)	0.100 *	(0.093, 1.806)	0.259 *	(0.008, 2.511)
Education (ref. = illiteracy)						
Primary school	0.076	(−0.415, 0.569)	−0.799	(−1.927, 0.329)	−0.242	(−1.823, 1.339)
Middle school or above	0.697	(−0.527, 1.922)	−0.179 *	(−5.512, −0.847)	−0.591*	(−8.848, −0.333)
Living in family house (ref. = yes)						
No	−0.769	(−2.182, 0.643)	−0.766	(−4.526, 0.993)	−0.924	(−5.796, 3.948)
Amount of social security (ref. = none)						
One	−0.043	(−0.425, 0.337)	0.537	(−0.257, 1.332)	0.172	(−1.180, 1.525)
Two or above	−0.642	(−1.487, 0.202)	−0.102	(−1.963, 1.757)	−0.274	(−2.547, 1.998)
Pseudo R^2^	0.213		0.242		0.216	

Note: * *p* < 0.05.

## Data Availability

The CHARLS data can be accessed through its official website (charls.ccer.edu.cn/en (accessed on 10 July 2021)).

## Supplementary Materials

The following are available online at https://www.mdpi.com/article/10.3390/healthcare9121765/s1. Supplemental Table S1 Definitions and variable construction of social determinants by domain
