# Supplementary material for: Multidomain Social Determinants of Depressive Symptoms for the Elderly with Chronic Diseases: Evidence from the China Health and Retirement Longitudinal Study
Source: Healthcare (Basel). 2021 Dec 20;9(12):1765. doi: 10.3390/healthcare9121765 (PMC8701898; doi:10.3390/healthcare9121765)
Supplement: Supplementary file 1 [file healthcare-09-01765-s001.zip › healthcare-1482342-supplementary.pdf]

Supplemental Table S1: Definitions and variable construction of social determinants, by domain

Domain	Variables	Measurements
**Demographic**: The specific demographic characteristics of populations that convey risk for, or protection from, mental illness	Gender (male/female)	Sex reported by respondents
	Age (integer)	Age reported by respondents
	Marital status (married/ others)	Current marital status reported by respondents
**Economic**: Factors relating to the production, consumption, and transfer of wealth that convey risk for, or protection from, mental illness	Annual income ($<$ 20000/ $\geqslant$ 20000)	Total income including wage, bonus, pensions, unemployment compensation and other types of income respondents received in the past year
	Working status (yes/no)	Did you work for at least one-hour last week
**Neighborhood**: Characteristics of a community that convey risk for, or protection from, mental illness, over and above what is attributable to the individual characteristics of community members	Residence (urban/rural)	Respondent's residential area
	Access to physical examination (yes/no)	Have you participated in the physical examination in the last two years
**Environmental events**: Serious disruptions of the functioning of a community that exceed its ability to cope by use of its own resources and convey risk for mental illness	Working age ($<$ 18/$\geqslant$18)	At what age did you start working (or farming)
	Disability experience (yes/no)	Do you have physical disabilities now or in the past
**Social and cultural**: ways in which the organization of society, social interactions, and relationships affect risk of, and protection from, mental illness	Education (illiteracy/ elementary school/ Middle school or above)	What's the highest level of education you have attained now
	Living in family house (yes/no)	Do you live in family house
	Number of social security (none/one/two or above)	Any social security the respondents were covered including retirement pension, public old-age insurance, commercialized old age insurance, public free medical services, medical insurance for urban workers and residents, the new rural cooperative medical insurance, commercial medical insurance and other
